# Finite Element Numerical Simulation of Deformation of Critical Vehicle Components and Damage to Retaining Walls of Emergency Escape Ramps During Truck Impacts

**DOI:** 10.3390/s25041013

**Published:** 2025-02-08

**Authors:** Pinpin Qin, Zhicheng Xu, Yiyuan Shi

**Affiliations:** Guangxi Key Laboratory of Manufacturing System & Advanced Manufacturing Technology, School of Mechanical Engineering, Guangxi University, Nanning 530004, China; 2211301076@st.gxu.edu.cn (Z.X.); 2311301054@st.gxu.edu.cn (Y.S.)

**Keywords:** emergency escape ramp, retaining wall, truck, crash analysis, finite element method

## Abstract

In this study, finite element numerical simulations were used to investigate the deformation of critical vehicle components and the damage characteristics of the retaining wall at the end of the emergency escape ramp after the impact of a Ford 800 truck on the retaining wall of the refuge lane. A finite element model of the reinforced concrete retaining wall of the truck was created using the LS-DYNA (R11.0) program and the correctness of the constructed finite element model was confirmed by tests. The parameters of the reinforced concrete retaining wall were determined using orthogonal tests. Finite element numerical simulations of vehicle impact on the retaining wall were carried out, and the results showed that two stages of deformation occurred at the front and rear sides of the cockpit during the impact process, and the damage of the retaining wall increased with the increase in the vehicle speed, the impact angle, and the bumper stiffness. Punching shear damage occurred in the impact region of the wall and shear damage occurred at the corners of the wall.

## 1. Introduction

An emergency escape ramp (EER) is a separate highway constructed outside of the main route in a continuous extended downhill portion to make the uncontrolled vehicle force braking and deceleration [1]. As the EER design is not standardized and the length of the brake bed cannot satisfy the requirement of halting the runaway vehicle, it is possible that this causes the runaway vehicle to run out of the EER, immediately fall into the cliff, and cause traffic accidents [2,3]. The uncontrolled vehicle’s stopping distance increases with the contaminated aggregate’s soil content [4]. Due to the lack of brake length, energy dissipation facilities are added at the end of the EER. Vehicle deformation and damage to energy dissipation facilities during accidents between vehicles and energy dissipation facilities are vital for safety and engineering construction, and these must be investigated.

The end energy dissipation facilities at the EER comprise energy dissipation drums, tires, and end-retaining walls. The National Center for Transportation Research (NCTR) and other organizations in the United States have developed vehicle finite element models for verifying roadway hardware analysis, and they have undertaken highway facility collision investigations using finite element simulation [5]. Qin et al. [6,7] proposed installing a fall arrest net at the end of the EER to catch the runaway vehicle. They utilized the finite element method to build and simulate the protection net to assess the fall arrest net’s dynamic response and safety performance under vehicle contact. Wambold J C et al. [8] investigated the efficacy of different layouts of energy-dissipating barrels for the interception of runaway vehicles. Qin et al. [9] investigated the influence of horizontally stacked and vertically stacked energy dissipation tires on the effectiveness of intercepting vehicles at the end of the EER, considering the blocking effect of the retaining wall. Still, the wall was only simulated with a rigid-body model. Studies have been conducted in the field of vehicle collisions with roadside infrastructure. Hu et al. [10] performed numerical simulations of vehicle impacts on collision avoidance columns. They adjusted the parameters of the collision avoidance columns by utilizing an orthogonal experimental design. It was revealed that the collision avoidance column’s height, diameter, and reinforcement strength considerably impacted the collision avoidance performance. Sharma et al. [11] evaluated the effect of several vehicles on concrete columns by using the finite element approach and examined the dynamic response and damage of concrete columns. Performance levels are created for distinct damage states, which enhance the dynamic analysis approach and may be expanded to estimate the load-carrying capability of additional parts. Yoshito Itoh et al. [12] utilized finite elements to simulate the impact of a heavy truck on a concrete barrier and showed the model’s reliability by comparing it to the experimental records of an actual full-sized vehicle. El Tawil et al. [13] established a model for diverse truck impacts on bridge abutments using LS-DYNA and performed simulation and real vehicle impact studies. The findings reveal that the short duration of the peak impact force is of little practical research importance, and the comparable static force is better suited to the design value of the impact force. Zhang et al. [14] used three concrete structural models, the concrete KCC model, the CSC model, and the KF model, to assess the accuracy of the numerical analysis of vehicle crashes with reinforced concrete bridge piers. Zhao et al. [15] used finite element (FE) simulation with LS-DYNA to investigate the dynamic behavior and damage mechanism of reinforced concrete (RC) bridge piers subjected to truck impact. It was concluded that the simulation technique using the FE model, material model, and contact algorithm can be used to study the impact process of RC members.

There are no crash studies on the retaining wall at the end of the EER, so the literature on the impact resistance of RC walls has been used as a reference. The concrete strength, impact velocity, and quality of reinforced concrete walls significantly affect their impact resistance, determining the magnitude of the peak impact force [16,17,18]. Özgür Anil et al. [19] investigated the effect of different support conditions on the impact resistance of reinforced concrete slabs. Ran Yang et al. investigated the behavior and damage evolution of RC walls under transverse impact using impact experiments and corresponding finite element models to quantify the impact resistance of different RC walls at various impact velocities and masses [20]. Arnold C. Y. et al. [21] conducted a study on RC impact barriers, evaluating and determining the design parameters of cantilevered RC walls. Duc-Kien Thai evaluated the impact resistance of RC walls under missile impact loading using the finite element method, which has proven to be useful in studying the punching behavior of RC walls and the vulnerability of different walls [22]. Yao, JC et al. [23] proposed a new type of assembled bridge guardrail and evaluated the impact effect of the vehicle and prefabricated bridge guardrails using the finite element method, and investigated the effects of vehicle mass, collision angle, initial speed, and collision point on the collision effect. Further research is necessary to fully understand the complicated process of vehicle contact on the end-retaining wall of the EER, including the deformation of the vehicle and the retaining wall’s resistance to impact. This research establishes a finite element model of a truck colliding with a reinforced concrete retaining wall. Reinforced concrete beam testing and finite element simulations proved the viability of the numerical modeling of the retaining wall impact. The structural parameters of the retaining wall were designed using orthogonal experiments. A numerical simulation of truck impact on the retaining wall was carried out with LS-DYNA to analyze the dynamic response of vehicle impact on the retaining wall and the damage to the retaining wall. In addition, the damage assessment and analysis of the wall under different working conditions were carried out using the average damage factor method.

## 2. Models and Methods

### 2.1. Finite Element Modeling of Mid-Sized Trucks

**Figure 1:**
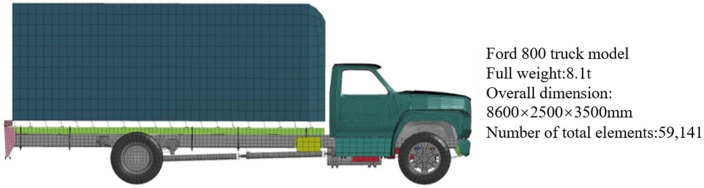
Ford 800 truck finite element model.

The vehicle model used in this paper is a Ford 800 (F800) mid-sized truck whose finite element model is shown in Figure 1. The finite element model of the Ford 800 truck was created by the National Crash Analysis Center (NCAC) at George Washington University and is jointly endorsed by the Federal Highway Administration and the National Traffic Safety Administration. The model is mainly used for the simulation and analysis of road safety facilities. The F800 truck consists primarily of an engine, body, chassis, and loaded cargo. The vehicle has a net weight of 5.27 tons and an overall mass of 8.1 tons. For detailed parameters, such as the materials and structure of the model, see reference [24]. Medium-duty trucks, when fully laden with goods, may result in collisions equally as serious and severe as those involving heavy-duty trucks [25]. The main reasons for choosing this model are as follows: the out-of-control vehicles driving into the EER are mainly large- and medium-sized trucks, and the F800, as a kind of medium-sized truck, has a certain degree of representativeness among the trucks; the F800 truck model has been widely used in the field of collision simulation research [13,26], and its validity has been verified by many scholars [27,28].

### 2.2. Establishment of Retaining Wall Finite Element Model

**Figure 2:**
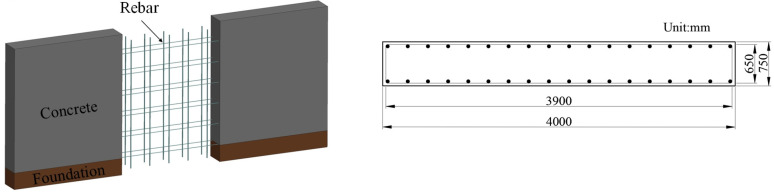
Concrete retaining wall configuration.

According to the industry standard “Design Details for Highway Emergency Escape Ramps” issued by China’s Ministry of National Transportation and Communications, the height of the retaining wall of the EER is not less than 1.5 m. It is constructed with reinforced concrete. In comparison, the width of the brake bed of the risk-avoidance lane is stated to be 4~6 m in the standard. Referring to the criteria in the standard, the end-retaining wall model established in this paper has the following three parts: concrete, rebars, and foundation. The configuration of the retaining wall is shown in Figure 2. The preliminary setting of the width of the retaining wall is 4000 mm, and the height of the retaining wall is 1500 mm. The longitudinal reinforcement and hoop reinforcement of the retaining wall are made of HPB235 grade steel; the diameter of the longitudinal reinforcement is 12 mm, the diameter of the stirrup is 6 mm, the spacing is 250 mm, and the reinforcing rate is 0.38%.

### 2.3. Validation of the Retaining Wall Model

Since it is difficult and dangerous to conduct experiments on the vehicle impact on reinforced concrete retaining walls directly with actual vehicles, an indirect validation approach is used to verify the validity of the finite element model. He et al. [29], in their study on the vehicle impact resistance of concrete frame structures, validated the material models of ordinary concrete and high-performance concrete using drop hammer impact-reinforced concrete beam experiments. The results show that the contact algorithm and material model used have good prediction accuracy for the damage and response of concrete frames upon impact, which can be used for numerical simulation of the impact damage of monolithic concrete frames.

In this paper, the validity of the finite element model of the retaining wall is verified by three-point bending experiments of reinforced concrete beams and simulation experiments performed with reference to the falling hammer impact on the reinforced concrete beams experiments conducted by Fujikake et al. [30], respectively.

**Figure 3:**
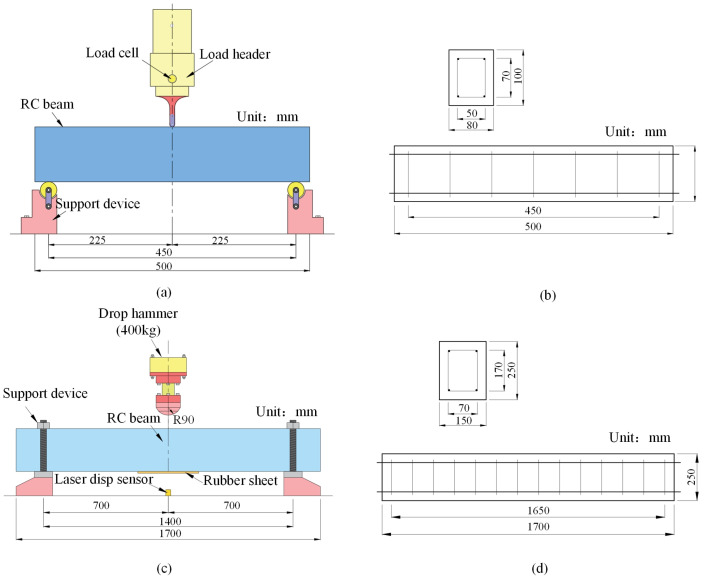
Experimental setup and beam configuration: (**a**) three-point bending experimental setup; (**b**) three-point bending beam configuration; (**c**) falling hammer experimental setup; (**d**) falling hummer test beam configuration.

A three-point bending loading was used to apply loads to the reinforced concrete beams in a linear static manner. The schematic of the experimental setup is shown in Figure 3a, and the dimensions of the reinforced concrete beam model are shown in Figure 3b. Fujikake et al. [30] conducted three experiments on reinforced concrete beams impacted by falling hammers under the following three working conditions: S1616, S1322, and S2222. In this paper, the reinforced concrete beams in the S1616 experimental condition were studied. For the S1616 working condition, the height of the drop hammer is 0.6 m, the speed of the drop hammer is set to 3.464 m/s, the total weight of the drop hammer is 400 kg in the simulation, and numerical simulations were performed to verify the validity of the reinforced concrete material model, simulation elements, contact types, and contact algorithms used in this paper. The detailed schematic diagram of the test setup is shown in Figure 3c, and the dimensions of the reinforced concrete beam model are shown in Figure 3d. The specific parameters of the reinforced concrete beam in the three-point bending experiment and the drop hammer experiment are shown in Table 1.

**Figure 4:**
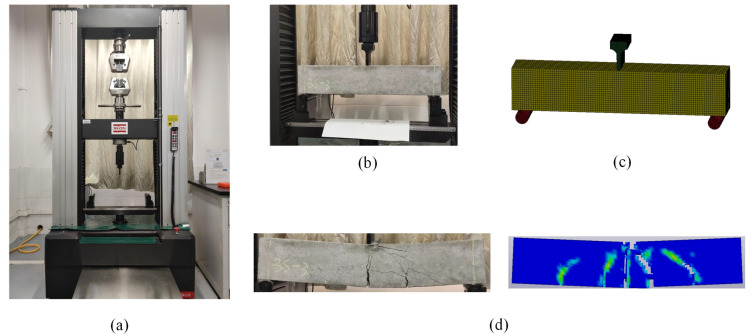
Three-point bending experimental equipment and results: (**a**) experimental equipment; (**b**) RC beam; (**c**) finite element modeling of concrete beams with three-point bending; (**d**) comparison of damage forms.

**Figure 5:**
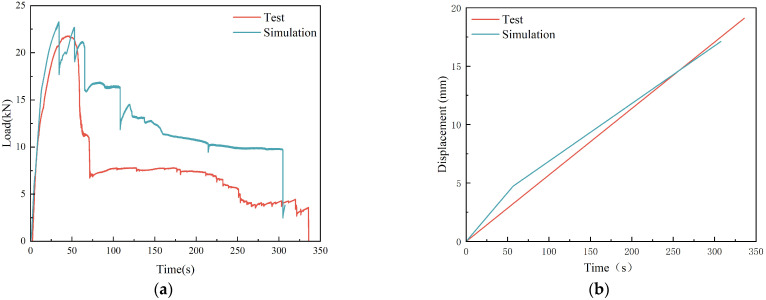
Response comparison of three-point bending experiments: (**a**) load curve; (**b**) mid-span displacement curve.

The three-point bending test of reinforced concrete beams was performed using static loading with a 5 mm/min loading rate. In LS-DYNA (R11.0) software, a finite element model was developed for the same working conditions, and the damaging cloud of reinforced concrete beams was calculated. A comparison of the results is shown in Figure 4, which shows that the damage patterns of the beams in the experiment and simulation are consistent. The time-dependent curves of the loads applied to the concrete beams and the mid-span displacements of the reinforced concrete beams are shown in Figure 5. The simulation results show that the load curves and mid-span displacements of the reinforced concrete beams are somewhat different from the experimental results, but the peak values and trends of the loads are the same. From the analysis above, it can be seen that the finite element model of the reinforced concrete beam is effective under static load and can be used later in the numerical simulation.

**Figure 6:**
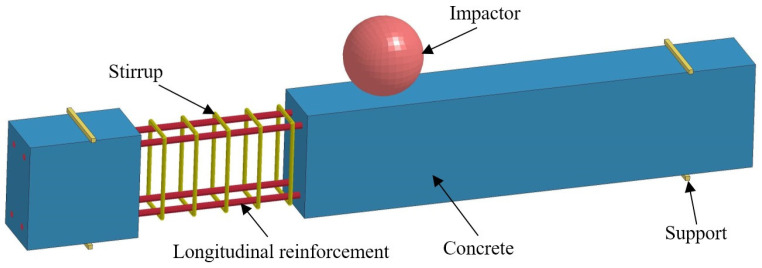
Finite element modeling of drop hammer-impacted reinforced concrete beams.

The cells, contact algorithm, contact type, and material model of the beams in the drop hammer impact-reinforced concrete tests are consistent with the finite element model of the reinforced concrete retaining wall. Both the support and the drop hammer were simulated using the material MAT_RIGID (rigid-body model), and the units were simulated using fully integrated solid units and constrained to the vertical displacements of the support. Numerical simulations were carried out using different concrete structural models to improve the accuracy of model validation. The concrete was simulated using the Continuous Surface Cap Model (CSCM) model, the KCC model, and the WINFRITH model for concrete. The material parameters are shown in Table 2, and the finite element model of the drop hammer and reinforced concrete beam are shown in Figure 6.

**Figure 7:**
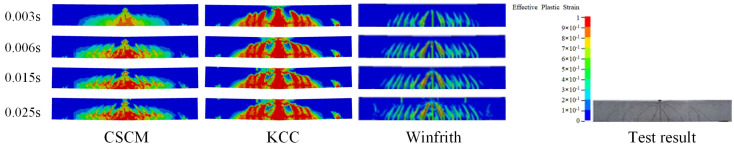
Comparison of damage and failure mode.

**Figure 8:**
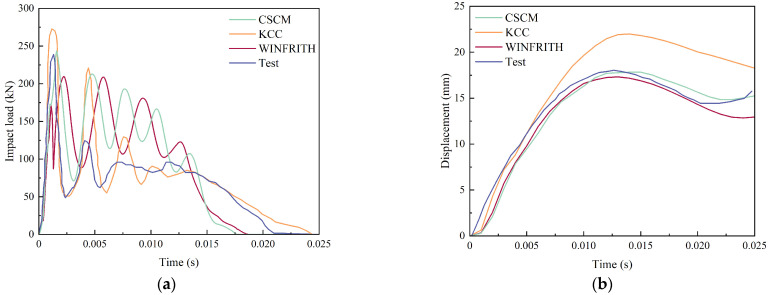
Comparison of dynamic response to drop hammer impact: (**a**) impact load-time course curve; (**b**) mid-span displacement curve.

The damage forms of reinforced concrete beams obtained after numerical simulation are shown in Figure 7 for the CSCM model, the KCC model, the Winfrith model, and the KCC model. The time course curves of the impact force of the falling hammer on the reinforced concrete beam and the deflection curves of the reinforced concrete beam are shown in Figure 8. By analyzing the three simulation models above, it is found that the damage patterns of the models are consistent with the experimental results in the literature [20]. This shows that the finite element model of reinforced concrete beams under impact loading is effective and can be used later in the numerical simulation.

### 2.4. Determination of Structural Parameters of the Retaining Wall

By comparing the results of experiments and simulations, it is found that the degree of destruction, damage, and the load range of the KCC model is much larger than the experimental results. The WINFRITH model can simulate the cracks well, but the apparent damage simulation needs to be more prominent. The CSCM model outperforms the other two models by simulating the vertical and diagonal cracks at the impact location very well, and the damage distribution is more uniform, with a peak error of only 2% in the impact force loading. Based on the above analysis, the concrete Continuous Surface Cap Model was selected for the retaining wall in this paper, developed by the U.S. Federal Highway Administration for the safety analysis of reinforced concrete parapets, with a material density of 2350 kg/m^3^. The foundations were simulated using a rigid material model (MAT_RIGID), and the vertical and horizontal displacements of the foundations were constrained. Reinforcing bars with a density of 7850 kg/m^3^ and a yield strength of 235 MPa were simulated using the elastic–plastic follow-hardening material model (MAT_PLASTIC_KINEMATIC).

Concrete and foundations are simulated using fully integrated solid units, and reinforcement is simulated using Hughes–Liu beam units. The coupling between reinforced concrete and concrete is realized by the Lagrange coupling algorithm and the keyword *CONSTRAINED_BEAM_IN_SOLID.

The crashworthiness of reinforced concrete retaining walls is affected by factors such as concrete strength, reinforcement ratio, thickness, and retaining wall height. The quadrature test method can handle multifactorial tests, and the number of tests and test time can be significantly reduced by designing the test conditions through the orthogonal test table [31]. Therefore, this paper uses the orthogonal test method to create the parameters of reinforced concrete retaining walls. As required by the Highway Emergency Escape Ramp Design Rules, the design variables for the orthogonal test are concrete strength (A), retaining wall reinforcement ratio (B), retaining wall thickness (C), and retaining wall height (D). The design parameters are shown in Table 3.

The kinetic energy absorption capacity of the retaining wall for vehicles, peak impact force, and average impact force were adopted as the evaluation indexes for the crashworthiness of the retaining wall. Among them, vehicle kinetic energy absorption is the primary reference index for the evaluation index of the crashworthiness of the retaining wall. The peak impact force represents the degree of damage to the vehicle and the retaining wall in the impact process; the smaller the value, the better the impact resistance. The average impact force represents the energy absorption capacity; the greater the value, the better the energy absorption capacity [32]. In the simulation test, the vehicle’s total mass was 8.1 t. The speed of an out-of-control vehicle before impacting the energy dissipation facility is generally less than 20 km/h [33]. It considers the situation of a short EER or a seriously speeding vehicle, and the vehicle’s speed before impact was set to 40 km/h.

Orthogonal experiments were based on the selected parameters of A, B, C, and D. A total of 16 simulation experiments were conducted and the related experimental data were obtained from LS-DYNA. The vehicle kinetic energy (absorb energy, AE) that can be absorbed by the retaining wall, the peak impact force during the collision (*F_max_*), and the average impact force (*F_ave_*) were used as evaluation indexes in the experimental design. The established L^16^ (4^4^) orthogonal experimental design table and calculations are shown in Table 4. Based on the different evaluation indicators, an analysis of variance was performed using the SPSS (22.0) statistical analysis software. The results show that a concrete strength of 40 MPa, a reinforcement rate of 0.95%, retaining a wall thickness of 750 mm, and a height of 2100 mm for energy absorption are optimal, wherein the primary and secondary factors are C > D > B > A; a concrete strength of 20 MPa, a reinforcement rate of 0.57%, retaining a wall thickness of 250 mm, and a height of 1700 mm are the levels at which the impact force peak value is the smallest, wherein the primary and secondary factors are A > C > D > B; a concrete strength of 40 MPa, a reinforcement rate of 0.95%, retaining a wall thickness of 750 mm, and a height of 2100 mm are the levels at which the average peak impact force is the smallest, wherein the primary and secondary factors are A > C > D > B.

**Figure 9:**
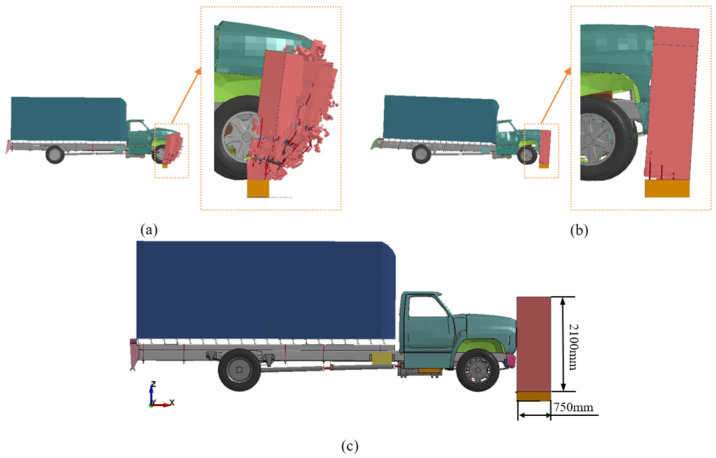
Finite element modeling of vehicle impacts on concrete retaining walls: (**a**) Experiment 1, retaining wall completely destroyed; (**b**) Experiment 5, retaining wall corner fracture; (**c**) Finite Element model for vehicle impacts on the retaining wall.

In the simulation tests, the kinetic energy of the runaway vehicle was 528 kJ. The reinforced concrete retaining walls failed to intercept the runaway vehicles in Experiments 1, 5, 6, and 11, of which Experiment 1 is shown in Figure 9a and Experiment 5 is shown in Figure 9b. The retaining walls appeared to be in overall fragmentation or the corners of the walls were broken. The reinforced concrete retaining wall could intercept the runaway vehicle in tests 2, 3, 4, 7, 8, 9, 10, 12, 13, 14, and 15. For reinforced concrete retaining walls, the ability to intercept runaway vehicles is the primary evaluation index. According to the evaluation results of other evaluation indexes, the optimal combination of a reinforced concrete retaining wall is determined as A3B4C3D4, which is the concrete compressive strength of 40 MPa, a reinforcement rate of 0.95%, a thickness of the retaining wall of 750 mm, and a height of the retaining wall of 2100 mm. The final finite element model of the runaway vehicle and the retaining wall is shown in Figure 9c.

## 3. Results

### 3.1. System Energy Change Analysis

**Figure 10:**
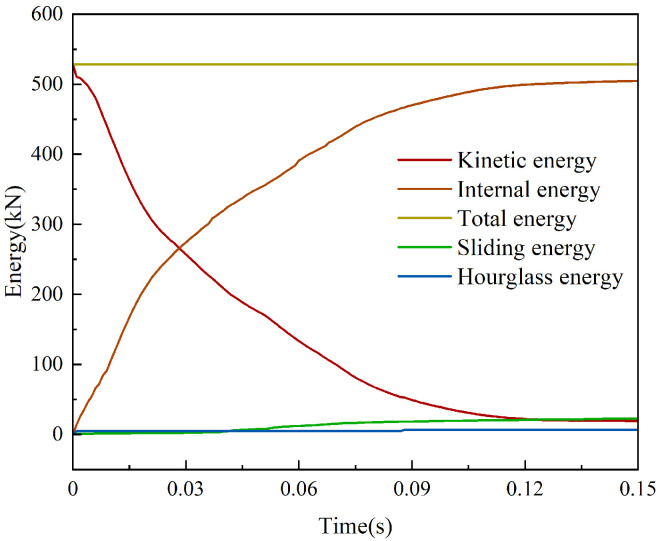
System energy changes.

The change in energy during the impact process is an essential basis for verifying the reasonableness of the numerical simulation. The time course curve of the energy change of the system during the impact is shown in Figure 10. The initial kinetic energy of the vehicle is 528 kJ, most of which is converted into the system’s internal energy. A small portion of the energy is converted into the slip and hourglass energy of the system. Combined with the finite element numerical simulation results, it can be found that when the vehicle collides with the retaining wall, the kinetic energy changes suddenly and decreases continuously. In contrast, the internal energy of the system starts to increase continuously. After the collision between the vehicle and the retaining wall is completed, the vehicle’s kinetic energy decreases to zero, the system’s internal energy no longer increases, and the kinetic and internal energy changes tend to level off. The model’s total energy remains unchanged, most vehicle kinetic energy is converted into system internal energy, and the hourglass energy can be controlled within 5% of the total energy. The analysis above shows the reasonableness of the model’s numerical simulation.

### 3.2. Vehicle Impact Process Analysis

**Figure 11:**
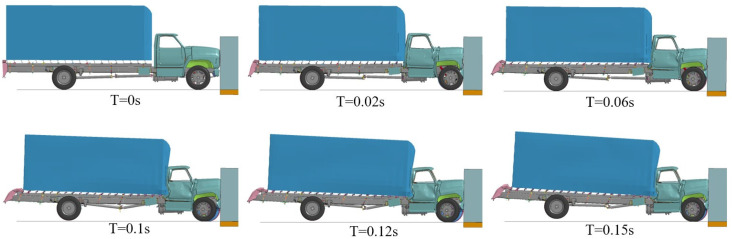
Numerical simulation process of truck hitting reinforced concrete retaining wall.

An analysis on how the numerical simulation process of the F800 truck impacted the reinforced concrete retaining wall was conducted; the impact process of A3B4C3D4 is shown in Figure 11. It can be seen that the vehicle impacts the retaining wall after T = 0 s. The hood of the vehicle first makes contact with the retaining wall, then the front bumper of the vehicle. The hood and the bumper are subject to elastic–plastic deformation during the collision. The container on the truck continues to move in a positive direction under inertia. Since the mass of the container constitutes a significant part of the vehicle’s mass, it generates a large amount of inertia. The truck’s cockpit was severely deformed under the crush of the container, and the front and rear enclosures and doors of the cockpit were bent and deformed to varying degrees. At T = 0.1 s, the truck’s rear wheels leave the ground under the effect of inertia, and the body presents a pitch angle of about 3° with the ground. Through the simulation results, it can be found that the vehicle chassis bears a more significant load, at T = 0.15 s. Under the extrusion of the cargo, the engine, fuel tank, and other parts connected to the chassis moved a certain distance to the ground, and the chassis had the “low in the middle and high on both sides” shape.

From the whole impact simulation process, it can be found that the main reason for the severe deformation of the truck’s cockpit is the extrusion of the cargo box on the cockpit under the impact of the vehicle speed of 40 km/h. It can also be inferred that when the vehicle speed is higher, the parts connected with the chassis may fall off, the cockpit will undergo more severe plastic deformation, and the survival space of the driver will be further narrowed, which poses a more severe threat to the safety of life.

### 3.3. Bumper Deformation Analysis

**Figure 12:**
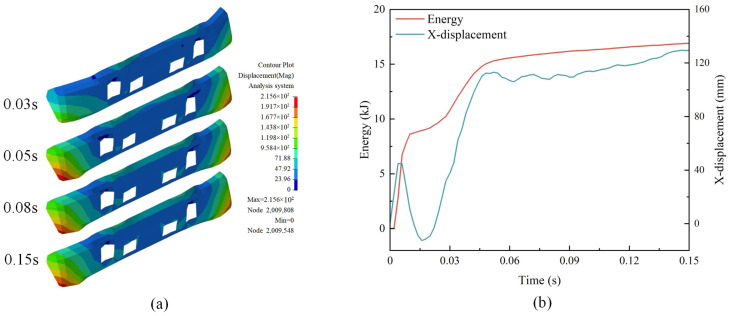
Bumper deformation process: (**a**) displacement of the front bumper; (**b**) energy change and displacement.

The truck’s bumper cushions when the vehicle hits the retaining wall. From Figure 12a, it can be seen that the bumper produced significant elastic–plastic deformation under the vehicle’s impact. The bumper deformation was evident in the period of T = 0~0.05 s. Initially, the bumper had a shape that was straight in the center and curved on both sides. At T = 0.05 s, the bumper became relatively straight due to the vehicle’s extrusion. After T = 0.05 s, the bumper’s shape does not change much. Figure 12b shows the variation of the bumper energy at different moments during the collision and the displacement of the outermost part of the bumper in the X direction. From the figure, it can be seen that the energy of the bumper changes less after T = 0.05 s, and this moment also corresponds to the deformation of the bumper. The bumper’s shape does not change much and stays mostly the same after T = 0.05 s.

### 3.4. Cockpit Deformation Analysis

**Figure 13:**
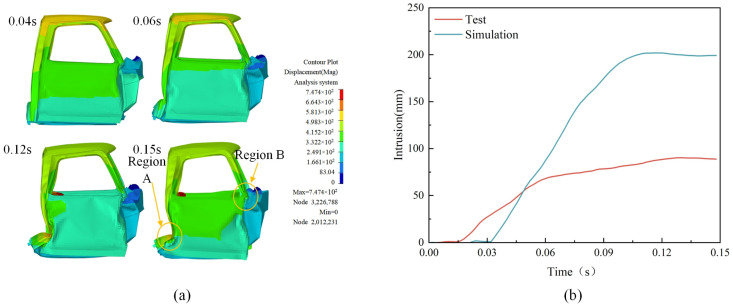
Cockpit deformation process: (**a**) cockpit displacement; (**b**) cockpit intrusions.

Significant deformations exist when the automobile collides with the retaining wall because the cargo box and the front of the car compress the cockpit. Figure 13a depicts the cockpit’s whole deformation process. Based on the figure, it is evident that the front side of the cockpit experiences the most distortion in T = 0~0.04 s. In contrast, the rear side experiences the most deformation after T = 0.04 s due to the container’s extrusion. The intrusion locations were mostly beneath the A-pillar of the cockpit’s front perimeter (Region A) and rear perimeter (Region B), according to an analysis of the intrusion volume in the cockpit. It is crucial to keep an eye on and gauge the intrusion volume; Figure 13b displays the intrusion volumes of the A and B areas. Region A is the point where the A-pillar meets the door, and Region B is the contact point between the cargo box and the rear enclosure of the cockpit. The intrusion data were obtained by LS-DYNA simulation. Based on the analysis, it is determined that T = 0~0.04 s is the stage of the vehicle and retaining wall collision, which causes a small amount of deformation on the cockpit; T = 0.04–0.1 s is the stage of cargo box impact, where a more significant amount of deformation is caused on the cockpit by the cargo box due to inertia; and T = 0.1–0.15 s is the stage of rebound, where the deformation of the cockpit tends to rebound.

### 3.5. Analysis of Vehicle Speed and Acceleration Changes

**Figure 14:**
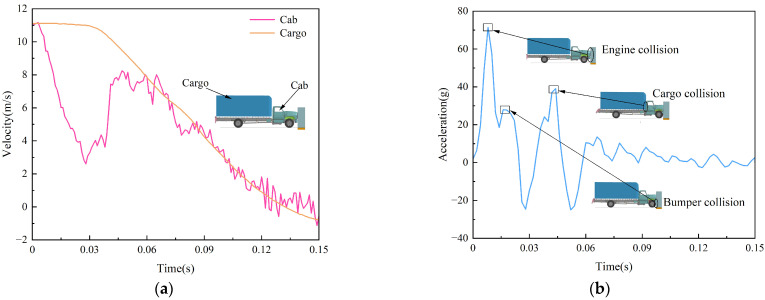
Vehicle speed and acceleration change curve: (**a**) velocity change curve; (**b**) acceleration change curve.

The cockpit and cargo box velocity time profiles for the F800 truck are shown in Figure 14a. It can be noticed that there is a difference in the speed change in different parts of the vehicle. Initially, the vehicle speed is 11.1 m/s, and T = 0~0.02 s is the collision stage between the vehicle and the retaining wall. At this time, the cockpit is the first to start decelerating, and the cargo box decelerates, lagging due to the role of inertia; T = 0.02~0.1 s is the inertial impact stage, the cockpit speed rises back up due to the inertial impact of the cargo box, and the cargo speed falls rapidly; and T = 0.1~0.15 s is the rebound stage, the vehicle structure recovers elastically after energy-absorbing deformation, and the vehicle speed drops to 0 and then rises to about 1 m/s in the reverse direction. The acceleration of vehicle collision has an essential impact on the safety of vehicle occupants, as shown in Figure 14b, which shows the acceleration of the cockpit area of the vehicle; it can be seen that a significant acceleration is generated at the moment the vehicle touches the retaining wall, and that the hood, bumper, and cargo box all cause an increase in acceleration during the impact with the retaining wall.

### 3.6. Retaining Wall Damage Change Process Analysis

**Figure 15:**
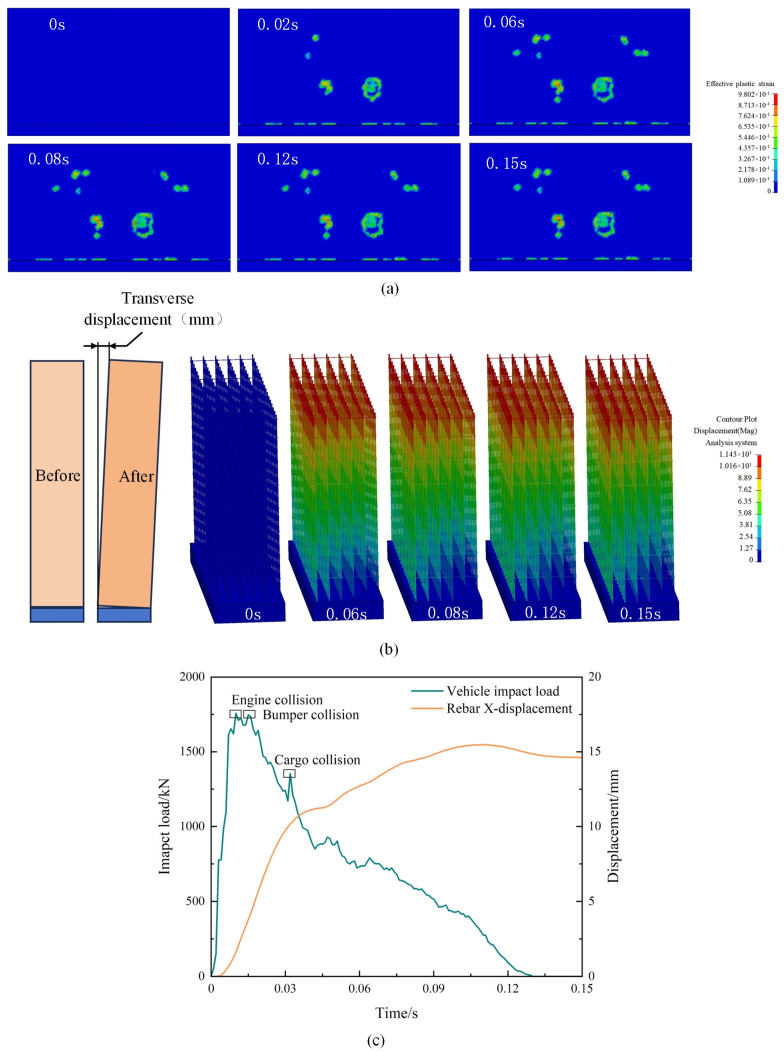
Results of retaining wall simulation: (**a**) retaining wall damage cloud map; (**b**) transverse displacement of reinforcement; (**c**) impact force and reinforcement deformation.

The frontal collision is used as a research object to analyze the damage to the retaining wall. The vehicle speed is 40 km/h. The damage change process of the reinforced concrete retaining wall is shown in Figure 15. It can be seen in Figure 15a that the damaged areas of the retaining wall were mainly found in the impact area of the vehicle and the retaining wall, and at the corners of the retaining wall. In the initial stage of impact (T = 0~0.02 s), the damage mainly occurred at a location about 800 mm from the bottom of the retaining wall, and this damaged area was also the impact location of the front bumper of the vehicle. Figure 15c shows that, at this time, the wall force body reached the first peak of 1758.2 kN; furthermore, the region of the local damage for the impact of vehicles and the retaining wall impact are caused by the impact area damage, the form of its damage for the impact shear damage, and the damage is more serious mainly manifested in the impact area of the concrete cracking and collapsing. In the first and middle stages of the impact (T = 0.02~0.08 s), the front-end components collided with the retaining wall, causing severe damage in the impact area. At T = 0.04 s, the wall force reached a second peak of 1354.4 kN due to the impact of the cargo box. The non-impact area was damaged to varying degrees under the action of the stress wave, and the damaged area was expanding. Meanwhile, the corners of the retaining wall were damaged due to the shear damage caused by the vehicle’s impact. It is observed through the cloud map that the damage to the wall corners is less severe, and the damage area is smaller. Figure 15b shows the cloud diagram of the change in the transverse displacement of the steel reinforcement of the retaining wall, and the transverse displacement of the retaining wall mainly occurs in T = 0~0.08 s, which is consistent with the stress of the retaining wall. In the middle and late stages of the impact (T = 0.08~0.15 s), the force of the retaining wall gradually decreases, the increment of the transverse displacement of reinforcement slows down, the damage area and damage degree of the concrete no longer increases, and the speed of the vehicle also decreases to zero at this stage.

## 4. Discussion

### 4.1. Parameterization for Different Working Conditions

Since there are different impact situations between the runaway vehicle and the retaining wall, the damage to the retaining wall under different conditions needs further investigation. Hence, collision experiments under various working conditions have to be carried out. Based on the determined structural parameters of the retaining wall, a simulation test was conducted on a reinforced concrete retaining wall impacted by an out-of-control vehicle with a total mass of 8.1 t and a speed of 40 km/h (control group). The speeds of the runaway vehicle were 5.5 m/s (20 km/h), 8.3 m/s (30 km/h), 11.1 m/s (40 km/h), and 13.8 m/s (50 km/h); the positions of the vehicle impacting the retaining wall with the centering offset distances were 0 mm, 200 mm, 400 mm and 600 mm; the angles of the vehicle impacting the retaining wall with the offset angles in the X direction are 0°, 4°, 8°, and 12°, respectively; and the stiffness of the front bumper of the F800 truck is 2 × 10^3^ MPa, 2 × 10^4^ MPa, 2 × 10^5^ MPa and 2 × 10^6^ MPa, respectively. The effects of the runaway vehicle’s speed, the vehicle’s position impacting the retaining wall, the vehicle’s angle impacting the retaining wall, and the stiffness of the vehicle’s front bumper on the dynamic response and damage to the retaining wall were investigated. In different working conditions, the parameter settings are shown in Table 5.

### 4.2. Analysis of Different Working Conditions

In structural design, the dynamic force is usually simplified to equivalent static force, which is the static force required to produce the same displacement corresponding to the dynamic load by applying a static load at the same point of action, and it depends on the dynamic properties of the impactor and the impacted object [34]. Therefore, in this paper, the equivalent static force of a vehicle impacting a retaining wall under different working conditions is calculated as follows, based on the global average method proposed in reference [34]:

(1) $$P_{t}=\frac{1}{t}\int_{0}^{t}F\left(t\right)dt$$

where F(t) refers to the time course of the impact force; $P_{t}$ refers to the equivalent static force; and t is the duration of the impact force.

The results of peak impact force, equivalent static force, lateral displacement of the retaining wall, and maximum stress in the reinforcement for different operating conditions are shown in Table 6. Table 6 shows that the vehicle speed significantly affects the peak impact force, equivalent static force, lateral displacement of the retaining wall, and maximum reinforcement stress. The peak impact force, equivalent static force, lateral displacement of the retaining wall, and maximum reinforcement stress tend to increase with increased vehicle speed. When the vehicle impacts the retaining wall with center offsets of 200 mm, 400 mm, and 600 mm, the different offset distances do not affect the vehicle’s peak impact force and equivalent static force. However, when the offset distance reaches 600 mm, the retaining wall’s maximum lateral displacement and the reinforcement’s maximum stress are greatly affected. When the vehicle impacted the reinforced concrete retaining wall at different angles, the peak impact force, equivalent static force, lateral displacement of the retaining wall, and the maximum stress of the reinforcement decreased with the increase in the impact angle, in which the lateral displacement of the retaining wall changed less. During the vehicle’s impact on the retaining wall, there is more contact between the vehicle’s bumper and the retaining wall. Therefore, the dynamic response of the bumper to the vehicle impact on the retaining wall was investigated for different moduli of elasticity (2 × 10^3^ MPa, 2 × 10^4^ MPa, and 2 × 10^6^ MPa). Compared to the control group of 2 × 10^5^ MPa, it was found that the peak impact force of the vehicle, the equivalent static force, and the maximum stress of the reinforcement all increased with the increase of the elastic modulus of the bumper. After the elastic modulus reached 2 × 10^5^ MPa, the effect of the increase in the elastic modulus on the vehicle peak impact force, equivalent static force, and maximum stress of reinforcement weakened, and the lateral displacement of the retaining wall did not change significantly during the change in the elastic modulus of the bumper.

### 4.3. Degree of Damage Under Different Working Conditions

In LS-DYNA, the damage factor is used to assess the degree of damage to the unit. The damage factor of the Continuous Surface Cap Model (CSCM) model is denoted by d, which varies from 0 to 1, reflecting the degree of damage of the concrete unit under impact loading. The larger its value is, the more serious the damage is; however, the evaluation object of this parameter is the component, and it is unreasonable to evaluate the overall damage to the concrete retaining wall. Zhao W et al. [35] suggest that the average value of the damage coefficient of members within the same cross-section should be used to evaluate the degree of damage to reinforced concrete beams. This evaluation method analyzes the degree of damage to reinforced concrete retaining walls. The formula for calculating the average value of the damage coefficient is as follows [35]:

(2) $$\bar{d}=\frac{\sum_{i=1}^{n}d_{i}}{n}$$

where $\bar{d}$ refers to the average damage factor in the region; i is the damage factor of the *i*th cell of the selected region; and n is the number of selection units.

According to Equation (2), the average damage coefficients of the impact zone of the retaining wall and the corners of the retaining wall under different working conditions are calculated, and the results are shown in Table 7. It can be seen that the vehicle speed significantly affects the damage to the retaining wall. With increased vehicle speed, the damage to reinforced concrete retaining wall impact zones and retaining wall corners becomes increasingly severe. The impact location had a negligible effect on the degree of damage in the impact zone. As the angle of impact increases, the degree of damage in the impact zone increases substantially, while the degree of damage at the corners of the retaining wall decreases. When vehicles with different stiffnesses of the bumper impacted the reinforced concrete retaining wall, the damage area of the retaining wall and the degree of damage at the corners of the retaining wall gradually increased with the stiffness.

## 5. Conclusions

This research has analyzed the process of an uncontrolled vehicle hitting the end-retaining wall of the emergency escape ramp, established a finite element model of a reinforced concrete retaining wall and verified its validity, determined the parameters of the retaining wall, and carried out a numerical simulation of a vehicle hitting the end-retaining wall of a refuge lane. The deformation of the critical components of the vehicle, the change of speed, and the influence of different working conditions on the damage of the retaining wall during the process of the medium-sized truck hitting the end-retaining wall of the emergency escape ramp were investigated, and the following conclusions were reached:

(1) When the concrete compressive strength of the retaining wall at the end of the EER is 40 MPa, the reinforcement rate of the steel bar is 0.95%, the thickness of the retaining wall is 750 mm, and the height of the retaining wall is 2100 mm, the retaining wall has a good collision resistance, which can achieve the effect of intercepting the out-of-control trucks, while the trucks gain a minor degree of damage in the collision process.
(2) During the impact process, the vehicle’s critical components’ deformation was divided into the front-end and cargo box impact stages. The vehicle’s deformation in the front-end impact stage was concentrated in the front bumper, hood, and front side of the cockpit; the cockpit’s deformation in the cargo box impact stage was more significant, mainly concentrated in the rear side of the cockpit.
(3) The peak impact force, the equivalent static force, the maximum lateral displacement of the retaining wall, and the maximum stress in the steel reinforcement all show an increasing trend as the vehicle speed increases. The effect of offset distance on the peak impact force, equivalent static force, and the dynamic response of the retaining wall of the vehicle is small. The peak impact force, equivalent static force, maximum lateral displacement of the retaining wall, and maximum stress of the reinforcement decreased with the increase in the impact angle. Different bumper stiffnesses have significant effects on the peak impact force, the equivalent static force, and the dynamic response of the retaining wall of the vehicle.
(4) When the vehicle is impacted by the reinforced concrete retaining wall, the damage area of the retaining wall is mainly concentrated in the impact area of the vehicle and the corners of the retaining wall, in which the damage of the impact area is more serious, belongs to the medium damage category—the form of destruction for the punching shear damage—and the corner of the region of the damage is lighter, belonging to the light damage category, the form of destruction for the shear damage.

## Figures and Tables

**Table 1:** Configuration parameters of reinforced concrete beams.

	Three-Point Bending Test	Falling Hammer Impact Test	Unit
Concrete compressive strength	30	42	MPa
Rebar yield strength	235	235	MPa
Longitudinal bars quantities	4	4	mm
Longitudinal bars diameter	12	16	
Stirrup diameter	6	10	mm
Stirrup interval	75	75	mm

**Table 2:** Simulation experiment material parameters.

Material	Material Model	Parameter	Value
CSCM	*MAT-159 (*MAT_CSCM_CONCRETE)	Density Compressive strength	2400 kg/m^3^ 42 MPa
KCC	*MAT-072R3 (*MAT_CONCRETE_DAMAGE_REAL3)	Density Compressive strength Poisson ratio	2400 kg/m^3^ 42 MPa 0.2
WINFRITH	*MAT-084/085 (*MAT_WINFRITH_CONCRETE)	Density Compressive strength Poisson ratio	2400 kg/m^3^ 42 MPa 0.2
Rebar	*MAT-03 (*MAT_PLASTIC_KINEMATIC)	Density Elastic modulus Yield strength Poisson ratio Failure strain	7800 kg/m^3^ 2 × 10^5^ MPa 426 MPa 0.3 0.12
Support	*MAT-020 (*MAT_RIGID)	Density	7800 kg/m^3^
Impactor	*MAT-020 (*MAT_RIGID)	Density	1330 kg/m^3^

**Table 3:** Retaining wall parameters design.

Factor/Level	A (MPa)	B (%)	C (mm)	D (mm)
1	20	0.38	250	1500
2	30	0.57	500	1700
3	40	0.76	750	1900
4	50	0.95	1000	2100

**Table 4:** Orthogonal test results.

Test Number	Performance Indicators			Test Number	Performance Indicators		
	AE/kJ	$F_{max}$/kN	$F_{ave}$/kN		AE/kJ	$F_{max}$/kN	$F_{ave}$/kN
1	35.01	1460.35	227.29	9	528.00	1771.29	850.49
2	528.00	1481.21	558.53	10	528.00	1781.14	831.74
3	528.00	1611.05	529.57	11	87.90	1612.08	339.65
4	528.00	1662.11	612.73	12	528.00	1721.18	639.94
5	376.36	1761.23	625.51	13	528.00	1700.06	587.04
6	99.64	1661.41	337.88	14	528.00	1651.21	579.49
7	528.00	1781.32	818.11	15	528.00	1621.11	563.45
8	528.00	1781.23	839.34	16	74.88	1531.18	253.40

**Table 5:** Parameters of simulated working conditions.

Working Condition	Parameters			
	Vehicle Speed (m/s)	Impact Position (mm)	Impact Angle (°)	Bumper Modulus of Elasticity (MPa)
C1-V20	5.5	0	0	2 × 10^5^
C2-V30	8.3	0	0	2 × 10^5^
C3-V40	11.1	0	0	2 × 10^5^
C4-V50	13.8	0	0	2 × 10^5^
C5-D000	11.1	0	0	2 × 10^5^
C6-D200	11.1	200	0	2 × 10^5^
C7-D400	11.1	400	0	2 × 10^5^
C8-D600	11.1	600	0	2 × 10^5^
C9-A0	11.1	0	0	2 × 10^5^
C10-A4	11.1	0	4	2 × 10^5^
C11-A8	11.1	0	8	2 × 10^5^
C12-A12	11.1	0	12	2 × 10^5^
C13-S3	11.1	0	0	2 × 10^3^
C14-S4	11.1	0	0	2 × 10^4^
C15-S5	11.1	0	0	2 × 10^5^
C16-S6	11.1	0	0	2 × 10^6^

**Table 6:** Dynamic response of retaining wall under different conditions of vehicle impacts.

Working Condition	Peak Impact Force (kN)	Equivalent Static Force (kN)	Lateral Displacement of Retaining Wall (mm)	Maximum Stress of Reinforcement (MPa)
C1-V20	1469.45	49.47	4.85	244
C2-V30	1668.12	73.76	8.08	256
C3-V40	1782.07	88.06	11.2	260
C4-V50	1825.07	101.13	15.1	275
C5-D000	1782.07	88.06	11.2	260
C6-D200	1786.80	97.59	11.1	259
C7-D400	1764.51	83.86	10.9	258
C8-D600	1801.33	97.87	18.1	295
C9-A0	1782.07	88.06	11.2	260
C10-A4	1900.38	98.11	10.7	255
C11-A8	1835.26	97.17	10.6	248
C12-A12	1786.78	77.17	10.1	240
C13-S3	1201.69	66.82	9.07	250
C14-S4	1731.54	85.79	10.3	258
C15-S5	1782.07	88.06	11.2	260
C16-S6	1789.71	93.39	11.5	260

**Table 7:** Damage results of reinforced concrete retaining walls.

Working Condition	Average Damage Factor $\bar{d}$		Working Condition	Average Damage Factor $\bar{d}$	
	$\mathrm{Impact}\;\mathrm{Area}\;\bar{d_{1}}$	$\mathrm{Corner}\;\mathrm{Area}\;\bar{d_{2}}$		$\mathrm{Impact}\;\mathrm{Area}\;\bar{d_{1}}$	$\mathrm{Corner}\;\mathrm{Area}\;\bar{d_{2}}$
C1-V20	0.409	0.157	C9-A0	0.519	0.232
C2-V30	0.452	0.194	C10-A4	0.476	0.257
C3-V40	0.519	0.232	C11-A8	0.483	0.215
C4-V50	0.588	0.301	C12-A12	0.610	0.258
C5-D000	0.519	0.232	C13-S3	0.469	0.183
C6-D200	0.455	0.243	C14-S4	0.490	0.199
C7-D400	0.448	0.249	C15-S5	0.519	0.232
C8-D600	0.446	0.251	C16-S6	0.583	0.242

## Data Availability

Data are contained within the article.
